# Quality early childhood education through self, workplace, or regulatory support: exploring the efficacy of professional registration for early childhood teachers in Australia

**DOI:** 10.1007/s13384-022-00575-8

**Published:** 2022-10-12

**Authors:** Marianne Fenech, Helen Watt

**Affiliations:** Sydney School of Education and Social Work, The University of Sydney, Sydney, Australia

**Keywords:** Teacher registration, Early childhood teachers, Critical policy sociology, Accountability, Early childhood education, Motivators and supports

## Abstract

Teacher registration is increasingly utilised as a governance mechanism to audit teachers’ work and drive professional practice. There is limited and mixed empirical evidence, however, as to whether registration drives teaching quality. Our study extends this limited empirical base by critically examining the policy trajectory in Australia to bring early childhood teachers into a uniform system of registration with primary and secondary teachers. Adopting a relatively novel methodology, the study intertwined a critical social policy framing with a national quantitative survey. Results showed that respondents perceived their professional self, followed by their workplace (colleagues and employer) as key influencers of quality practice, and neither agreed nor disagreed that teacher registration was beneficial. Findings problematise the need for, and benefits of, teacher registration. That early childhood teachers’ practice and development was most driven by intrinsic motivation and, to a lesser extent, being employed in high-quality, not-for-profit, and preschool settings where other early childhood teachers are employed, suggests that more effective and progressive policy approaches to support quality early childhood education require an addressing of the contexts and conditions in which early childhood teachers work.

## Introduction

As government investment in early childhood education (ECE) in Australia and internationally has grown, so too have accountability standards been increasingly adopted as a policy mechanism to assure and improve the quality of ECE provided (Grieshaber, 2017). The reach of this standards movement has included early childhood curriculum, the regulation of ECE services and teacher training, and the professional registration of early childhood teachers (ECTs). As a policy lever to support and ensure teacher quality, teacher registration in Australia aligns with shifts to professionalise the ECE workforce (Pascoe & Brennan, 2017).

Globally, governments have positioned teacher quality across all education sectors as a policy problem that warrants increasing and new modes of governance (Green et al., 2017; Rizvi & Lingard, 2010). The auditing of teachers’ work and professionalism through teacher registration and associated teaching standards has emerged as one such governance mechanism (Call, 2018; Havnes, 2018; Organisation for Economic Co-operation & Development [OECD], 2013; Révai, 2018; Toledo-Figueroa et al., 2017) and is indicative of a managerial approach that dictates professionalism from above (Havnes, 2018; Oberhuemer, 2005). Critics of the increasing implementation of accountability technologies such as teacher registration have problematised this policy trajectory for its erosion of teacher autonomy, control of teachers’ work, and confining of teachers’ practice to top-down, measurable technical competencies (Havnes, 2018). In these ways, the professionalism of teachers is considered to be diminished from both governance and epistemological positionings (Havnes, 2018; Holloway & Brass, 2018).

To date, however, few empirical studies have focused on how these conceptual criticisms of increasing teacher surveillance and accountability play out at the policy implementation stage (Garver, 2019). Indeed, the perspectives of teachers on these issues is under-researched, thereby limiting teachers’ input into policy debates relevant to their work (Anagnostopoulos et al., 2021). With regards to teacher registration, there is limited and mixed empirical evidence nationally and internationally to support the claim that it drives teacher quality and improves student outcomes (Call, 2018; Toledo-Figueroa et al., 2017). Studies suggest that the lived experience of teacher registration can engender effects that are deleterious to its purported intent, for example, by adding to teachers’ administrative workload burden; by constraining their practice; and by conveying a deficit image of teachers through a positioning of teaching quality as a problem that requires addressing through regulation.

In the Australian schools context, the meeting of professional teaching standards has been criticised as time-intensive professional compliance and surveillance, rather than a policy lever that supports authentic professional development (Bourke et al., 2015; Talbot, 2016). Consistent with international studies, concerns raised over the specificity of registration requirements include a diminution of professional autonomy and the deflection of attention away from structural barriers to quality education, to individual teachers (Chatelier & Rudolph, 2018). In turn, these deleterious effects are said to generate a societal mistrust of teachers and narrowing of practice, including professional development, to technicist, performative work.

In the context of ECE, only one small-scale qualitative study (Fenech et al., 2021) has focused on the registration of teachers in Australia who work in the ECE sector with children aged birth to 5 years.^1^ Focusing on the experiences of three ECTs, this study problematised the need for, and government-purported benefits of, teacher registration. For these participants, teacher registration compromised professional practice, job satisfaction, and provision of quality ECE.

The present study extends this limited empirical base by critically examining the policy trajectory in Australia to improve teacher quality and status by bringing all ECTs into a uniform system of teacher registration with primary and secondary teachers. We begin by providing an overview of teacher registration reform in Australia as a quality assurance policy lever for the ECE sector. We then present findings from our national survey that investigated ECTs’ perceptions of the efficacy of teacher registration, potential policy options, and other practice motivators and supports for ECE workforce quality, development, and status in Australia. We conclude by discussing implications of our findings for future policy directions, including consideration of standards, service, and self-governance of ECTs in the context of an impending national teacher registration system.

## Professional registration reform for ECTs in Australia

While in Australia professional registration for primary and secondary teachers is well established, the registration of ECTs—particularly those employed in non-school settings with children in the birth-5 years age group—is more piecemeal. As outlined in Table 1, three different policy approaches to ECT registration are in place across Australia’s eight state and territory jurisdictions. One approach is that all ECTs are required to register, irrespective of whether they are employed in a school or other ECE setting (South Australia, Western Australia, New South Wales, and Victoria). A second approach requires only ECTs employed in schools to be professionally registered (Australian Capital Territory, Tasmania, and the Northern Territory). In the third approach, professional registration is compulsory for ECTs employed in schools but voluntary for only some ECTs working in non-school settings; specifically, those employed to deliver an educational programme to children in the year before school and who meet prescribed registration requirements, including the meeting of the APST (Queensland). ECTs employed in long day care in Queensland do not meet these requirements (unless they are primary-qualified), and therefore are not eligible for professional registration. Through these varying approaches, the majority of ECTs in Australia today are professionally registered (Education Services Australia, 2018).

**Table 1:** Requirements for early childhood teacher registration across Australia (ACECQA, n.d.; Education Council, 2019)

	Jurisdiction	Registration requirements for early childhood teachers working with children birth—5 years
**APPROACH 1**	South Australia (SA)	All ECTs registered since 1976
	Western Australia (WA)	All ECTs employed in nationally recognised services registered since 06 December 2012
	New South Wales (NSW)	All ECTs employed in nationally recognised services registered since 18 July 2016 (referred to as teacher accreditation)
	Victoria (VIC)	All ECTs registered since 30 September 2015, but on a separate register from primary and secondary teachers
**APPROACH 2**	Australian Capital Territory (ACT)	Only ECTs employed in schools are registered (preschools for children 3–5 years are part of the school sector)
	Tasmania (TAS)	Only ECTs employed in schools (kindergartens for children 4–5 years are part of the school sector)
	Northern Territory (NT)	Only ECTs employed in schools (N.B. preschools for children 4–5 years are part of the school sector)
**APPROACH 3**	Queensland (QLD)	Compulsory registration for ECTs employed in the school sector (prep for children in the year before school is part of the school sector) Voluntary registration for ECTs employed in non-school settings who deliver an education programme to children in their pre-prep year

In response in part to the disparate approaches to registration for ECTs, in 2017 the Council of Australian Government’s Education Council established terms of reference for a National Review of Teacher Registration, to be undertaken by the Australian Institute for Teaching and School Leadership (AITSL) (Education Services Australia, 2018). This Review was preceded by a developing national approach to teacher registration, which began in 2011 with the National Framework for Teacher Registration. The Framework stipulated that in all jurisdictions, teacher registration would apply only to teachers employed in schools (Education Services Australia, 2018). It also established the Australian Professional Standards for Teachers (APST) (Education Services Australia, 2011) that were to be embedded into each jurisdiction’s system of teacher registration. Graduate teachers are awarded initial provisional registration but are required to demonstrate proficient meeting of the APST in their practice to attain full registration. Teachers may progress to the highest ‘Highly Accomplished’ and ‘Lead’ stages of registration by meeting the APST at these respective advanced levels. The 2018 Review was also preceded by a case study evaluation of the APST in schools but not prior-to-school services (AITSL, 2016). This evaluation concluded that the APST facilitated pre-service and graduate teachers’ practice, fostered cultures of learning, and assisted teachers’ professional development and career progression.

The 2018 Review’s terms of reference focused on the development of the National Framework and teacher registration as a mechanism to drive teacher quality in prior-to-school and school contexts (Education Services Australia, 2018). Following 140 consultation meetings with key sector stakeholders, 94 written submissions, and responses to an online survey from 6500 teachers, two of the subsequent 17 recommendations were specific to ECTs employed in prior-to-school services (Education Services Australia, 2018). Recommendation 5 maintained that “all ECTs in Australia, regardless of their employment setting, be required to be registered by teacher regulatory authorities, under a consistent national approach” (p. iv). Recommendation 6 acknowledged an orientation of the APST to primary and secondary education, proposing that “the Teacher Standards be amended to ensure their relevance and applicability to ECTs” (p. v). Other recommendations relevant to early childhood initial teacher education programmes or to ECTs employed to work with children birth-5 years included: Recommendation 8, that initial teacher education programmes include a greater focus on the APST and registration requirements; Recommendations 9, 10, 12 and 13, that information transfer across jurisdictions be streamlined to strengthen children’s safety, improve teacher employability, and support teaching as one profession; and Recommendation 14, that the national approach to English proficiency assessments be revised. The Review also identified issues specific to ECTs employed in the ECE sector that any proposed implementation plan would need to address: that an ECT is often the only qualified teacher employed in a prior-to-school setting; that there is a lack of onsite or organisational mentors to support ECTs’ transition from provisional to proficient registration; that ECTs are already accountable to, and provide evidence for education quality through the National Quality Framework (NQF)^2^ (Australian Children’s Education and Care Quality Authority [ACECQA], 2020), and that ECTs have limited access to quality professional development. While the Review’s recommendations recently received ministerial endorsement (Education Council, 2019), the COVID-19 pandemic paused progress on their implementation.

Importantly, these recommendations were based on input from ECE sector stakeholders, including submissions from early childhood peak organisations, regulatory authorities and providers, and responses from 878 ECTs and early childhood leaders to the Review’s online survey (13% of the survey’s total 6569 teacher/leader respondents). Findings from the survey suggested broad support for teacher registration. For example, 78% of ECT respondents agreed that teacher registration is worthwhile; 68% could see the relevance of the APST to their practice; and 83% of early childhood directors agreed that registration supported the professional recognition of ECTs.

These findings, however, need to be considered in the context of the Review’s consultative process which discursively positioned teacher registration as necessary for teacher quality. For example, input was sought on *how* rather than *if* the APST has supported teacher quality, and *how* rather than *if* a nationally consistent approach to teacher registration would support and improve quality teaching. Similarly, some reporting of survey findings appeared skewed in favour of teacher registration. For example, although 78% of 600 ECT survey respondents considered teacher registration ‘worthwhile’ (Education Services Australia, 2018, p. 73), fewer than half these respondents agreed that registration supports the professional recognition of teachers and contributes to improving teachers’ professional practice. Moreover, and consistent with primary and secondary teacher respondents, ECT respondents did not rate the utility of the APST highly, with below midpoint scale scores on the importance of aligning their practice and maintaining proficiency against the Standards (respectively scored 3.12 and 3.11 out of 7). Greatest support for teacher registration appeared to be on compliance grounds, with respondents most strongly agreeing that only registered teachers should secure teaching employment and ensure the protection of children. Qualitative survey data reflected concerns about nationally inconsistent approaches to registration, associated regulatory burden, the orientation of the APST to primary and secondary teaching, and the need for improved access to quality professional development opportunities for ECTs and mentors to support graduate teachers’ transition to full proficiency registration (Education Services Australia, 2018).

In the section that follows we report on our empirical investigation of ECTs’ perceptions of the efficacy of professional registration in Australia. The study contextualised ECTs’ views about, and experiences of, teacher registration with an exploration of other perceived enablers of professional development and improvement of practice, taking into account characteristics of their workplace setting. Findings challenge dominant, taken-for-granted discourses about teacher registration as a necessary, or beneficial, policy lever to drive teacher quality and outcomes for children.

## Exploring the efficacy of professional registration for ECTs in Australia

### Conceptual approach

We situated our study in critical policy sociology (Ozga, 2021), which, in the context of education, problematises neoliberal, managerial positionings of teacher-professionalism (Ball, 1994, 2016). Following Foucault (1984), to ‘problematise’ means to critically consider how an object or concept like ‘teacher quality’ has come to be constructed as a problem, and how purported truths and solutions like ‘teacher registration’ reinforce the problem construction. Problematisation is fundamental to critical policy sociology (Ozga, 2021).

From this orientation we adopted a deliberatively interrogative stance to the governing of teachers’ work and the discursive framing of teacher registration as a solution to the policy-constructed teacher-quality problem. Accordingly, we explored the politics of this policy lever, for example, ECT participants’ perceptions of the discursive truth claims about teacher registration purported in the 2018 Review, and in the context of participants’ own professional development, whether registration technologies produced ‘policy subjects’ (Braun et al., 2010).

While we reject the adoption of a positivist ontology to policy research, our approach embraced Salomon’s (1990) seminal invitation for researchers to complement a selected paradigm with epistemological approaches traditionally considered to belong outside of its parameters. We therefore utilised a quantitative survey to “cohabitate” (Salomon, p. 16) our critical social policy framing of the study, and imbued discussion of our quantitative results with critical policy sociology interpretations.

### Participants

In total, 463 ECTs (416 registered and 47 non-registered) working with children birth-5 years completed the online survey. The selected age-range of registered participants was on average ‘41–45 years’ of age^3^ (*SD* = 2.17; range 18–25 years, to over 60). Average length of registration was 5.53 years (SD = 6.81; range 1 month-42 years, seven missing), and average employment in ECE was 14.61 years (*SD* = 10.50; range 1 month-44 years). In their current workplace, registered ECTs worked with an average of 2.89 other ECTs (*SD* = 1.72; range 1–10). Excluding the 79 registered ECTs who were the sole ECT at their workplace, other registered participants (*n* = 337) reported working with an average of 3.34 ECTs (SD = 1.61). Registered participants worked in centres licensed for an average of 60.02 children per day (*SD* = 32.20; range 19–220, 28 missing). Unregistered ECTs were also 41–45 years of age on average (*SD* = 2.11), had been employed in ECE for 10.42 years (SD = 10.06; range 1 month-46 years), and worked with an average of 2.34 other ECTs (*SD* = 1.46; range 1–7). Excluding the 13 unregistered ECTs (*n* = 34) who were the sole ECT at their workplace, other unregistered participants reported working with an average of 2.85 ECTs (SD = 1.42). Unregistered participants worked at centres licensed for an average of 67.91 children per day (SD = 26.60; range 24–128, missing 4). Table 2 depicts the age range of the sample, their ECT qualification and registration status, state/territory and location of the service in which they worked, their service type and National Quality Standard (NQS) rating, age group primarily worked with, provider type, and management structure.

**Table 2:** Demographic information of registered and unregistered ECT survey respondents (*N* = 463)

	Registered		Unregistered	
	*N*	%	*N*	%
*Age*				
18–25	12	2.9	1	2.1
26–30	43	10.3	4	8.5
31–35	55	13.2	8	17.0
36–40	56	13.5	8	17.0
41–45	66	15.9	7	14.9
46–50	63	15.1	8	17.0
51–55	50	12.0	4	8.5
56–60	44	10.6	5	10.6
Over 60	27	6.5	2	4.3
*Qualification*				
Birth to five	168	40.4	33	70.2
Birth to eight	159	38.2	8	17.0
Birth to twelve	74	17.8	5	10.6
Other	15	3.6	1	2.1
*Current registration level*				
Not registered	–	–	47	100
Conditional or provisional	69	16.6	–	–
Proficient	314	75.5	–	–
Highly accomplished	17	4.1	–	–
Lead teacher	16	3.8	–	–
*State/territory*				
Queensland	39	9.4	12	25.5
New South Wales	260	62.5	13	27.7
Victoria	69	16.6	6	12.8
South Australia	31	7.5	2	4.3
Australian Capital Territory	7	1.7	6	12.8
Western Australia	4	1.0	0	0
Tasmania	3	0.7	6	12.8
Northern Territory	3	0.7	2	4.3
*Location*				
Metropolitan	272	65.4	34	72.3
Rural	144	34.6	13	27.7
Remote	0	0.0	0	0.0
*Service type*				
Preschool/kindergarten	201	48.3	9	19.1
Long day care service	186	44.7	33	70.2
Other [inc. school]	29	7.0	0	0.0
Missing	0	0.0	5	10.6
*National Quality Standard (NQS) rating*				
Not yet rated	0	0	4	8.5
Working towards NQS	39	9.4	4	8.5
Meeting NQS	139	33.4	11	23.4
Exceeding NQS	202	48.6	28	59.6
Excellent NQS	4	1.0	0	0
Missing	32	7.7	0	0.0
*Age group worked with*				
Birth to 2 years	12	2.9	4	8.5
Two to three years	23	5.5	1	2.1
Three to five years	308	74.0	22	46.8
Birth to 5 years	37	8.9	9	19.1
Non-teaching ECT	36	8.7	11	23.4
*Provider type*				
Stand-alone	224	53.8	22	46.8
One of up to 10 managed by the same provider	67	16.1	10	21.3
One of up to 25 managed by the same provider	26	6.3	3	6.4
One of more than 25 managed by the same provider	98	23.6	12	25.5
Missing	1	0.2	0	0.0
*Management structure*				
For-profit	101	24.3	18	38.3
Not-for-profit, community managed	315	75.7	29	61.7

### Data collection

Following ethics approval from the researchers’ University Human Research Ethics Committee (Approval no. 2019/698), a national survey was administered online through the Qualtrics survey platform from late September until the end of November 2019, approximately 1 year after the release of the National Review report (Education Services Australia, 2018). The survey was promoted via ECE providers, peak organisations, unions, regulatory bodies and ECT social media sites.

The survey comprised four main sections: demographic information about the ECT respondents and the ECE service in which they were employed; perceived motivators and supports of their professional development and practice improvement (10 items); perceived benefits of teacher registration (15 items); and perspectives on a range of potential teacher registration policy directions, including recommendations from the National Review and how a national system of teacher registration might be operationalised (10 items). Participants responded to rating items on a five-point Likert-type scale: 1 = Strongly Disagree, 2 = Disagree, 3 = Neither Agree nor Disagree, 4 = Agree, and 5 = Strongly Agree. Items were developed by the lead researcher, informed by previous research (Fenech et al., 2021), claims made in the National Review, and the paper’s conceptual framework.

## Findings

Findings specific to 416 registered ECT respondents’ (i) motivators and supports of their professional development and practice improvement, (ii) perceived benefits of teacher registration, employment contexts, and (iii) preferred future policy directions given the National Review’s recommendation that AITSL proceed with the development of a national system of registration inclusive of ECTs, are presented in turn. The group of 47 unregistered ECTs was compared on their preferred future policy directions to explore potential differences for registered versus unregistered ECTs. Low missing data were excluded listwise per analysis; mean scores were interpreted in relation to the nearest scale point anchor.

### Specific motivators and supports of registered ECTs’ professional development and practice improvement

One of the purported benefits of teacher registration is that it does and will improve teacher quality. Our findings did not support this claim in terms of ECTs’ perceived professional development and practice improvement. As reported in Table 3, across 10 items, registered ECT respondents rated regulatory requirements as providing the least motivation and support for their professional development and practice improvement. The specific regulatory process of teacher registration was rated lowest of all, on average at the scale midpoint “Neither agree nor disagree”. In other words, responses on average from registered ECT respondents did not reach an “Agree” rating. Respondents identified their own professional self (that is, “passion and commitment to high quality ECE; intrinsic drive for quality improvement”) overwhelmingly as what most motivated and supported their development and practice as teachers, on average “Strongly agree”. Centre-specific motivators and supports—fellow educators and employers—were perceived to provide the next highest level of motivation and support for ECTs’ development and improved practice, on average “Agree”.

**Table 3:** Practice motivations and supports by currently registered ECTs: Factor loadings and descriptive statistics

	*Rotated factor loadings*			*Descriptive statistics*	
	Workplace (α = .81)	Requirements (α = .78)	Self (α = .67)	*M*	*SD*
The educators you work with support your development as a teacher	**.66**	.06	.17	3.79	1.00
The educators you work with motivate you to improve your practice	**.64**	.16	.09	3.88	1.04
Your employer supports your development as a teacher	**.63**	.20	.09	3.91	1.15
Your employer motivates you to improve your practice	**.60**	.02	.19	3.68	1.10
The process of attaining/maintaining teacher registration supports you to improve your practice	.10	**.67**	.08	3.24	1.12
The process of attaining/maintaining teacher registration motivates you to improve your practice	.10	**.64**	.06	3.14	1.19
The NQF and its assessment and rating process support you to improve your practice	.16	**.55**	.25	3.34	1.11
The NQF and its assessment and rating process motivate you to improve your practice	.07	**.50**	.27	3.57	1.12
Your professional self supports your development as a teacher (e.g., passion and commitment to high quality ECE; intrinsic drive for quality improvement)	.12	.21	**.44**	4.60	0.75
Your professional self motivates you to improve your practice (e.g., passion and commitment to high quality ECE; intrinsic drive for quality improvement)	.22	.11	**.42**	4.57	0.93

*NQF* National Quality Framework

Image factoring with varimax rotation converged in 5 iterations; listwise *N* = 394

Bold indicates highest loadings

All items were rated on a 5-point scale from 1: Strongly disagree, 2: Disagree, 3: Neither agree nor disagree, 4: Agree, 5: Strongly agree

### Specific benefits of teacher registration

To examine specific perceived benefits of teacher registration for ECE, we analysed registered participants’ responses to 15 items. The only item that was on average endorsed (i.e., rated close to 4 “Agree” on the scale), was the negative statement, “Having to attain and maintain professional teacher registration adds to regulatory burden because of the paperwork involved”. The only item that was “disagreed” on average, was that “Having to attain and maintain professional teacher registration has led to an increase in my salary”. On average, there was neither agreement nor disagreement from respondents that teacher registration improved examined outcomes including the quality of ECE services, the quality of respondents’ practice, outcomes for children, and the status of ECTs (see Table 4).

**Table 4:** Perceived benefits of teacher registration by currently registered ECTs: Factor loadings and descriptive statistics

	Factor loadings	Descriptive statistics	
	Benefits (α = .93)	M	SD
Having a system of teacher registration has helped improve outcomes for young children	.82	3.22	1.14
Having a system of teacher registration has helped improve the quality of my teaching practice	.81	3.02	1.11
Having a system of teacher registration has helped improve the quality of ECEC services	.81	3.14	1.14
Having a system of teacher registration has helped improve the status of early childhood teachers	.72	3.29	1.26
*Having to attain and maintain professional teacher registration:*			
…has increased my job satisfaction	.80	2.79	1.06
…has improved outcomes for the young children I work with	.79	3.04	1.12
…has supported my professionalism as a teacher	.78	3.46	1.03
…has meant that the level of quality ECEC provided at my service has improved	.77	2.93	1.03
^a^…is just a ‘tick the box’ exercise that I do so that I can keep practising as an ECT	−.68	3.07	1.20
…has increased recognition of the professional work that I do	.64	2.69	1.25
^a^…has decreased my job satisfaction	−.56	2.82	0.97
…means that my employer provides access to PD so that I can meet professional learning registration requirements	.55	3.12	1.21
^a^…is more about quality control than genuine quality improvement	−.53	3.27	1.04
…has led to an increase in my salary	.47	2.00	1.03
^a^…adds to regulatory burden because of the paperwork involved	−.47	3.73	1.02

*PD* professional development

Image factoring; listwise *N* = 402

All items were rated on a 5-point scale from 1: Strongly disagree, 2: Disagree, 3: Neither agree nor disagree, 4: Agree, 5: Strongly agree

^a^Items reversed when creating factor score

### General motivators and supports of registered ECTs’ professional development and practice improvement

It was of interest to explore whether the 10 items concerning registered ECTs’ perceived motivators and supports for professional development and practice improvement tapped into larger underlying constructs. An exploratory factor analysis (image extraction with varimax rotation) supported three latent constructs based on the scree-plot criterion and conceptual interpretability, explaining 38% of the total variance. All items had their primary loading on one of the three factors with no concerning cross-loadings. We named the first factor ‘Workplace’ (α = 0.81), containing 4 items pertaining to “educators you work with” and “your employer”. The second factor was named ‘Requirements’ (α = 0.78), containing the 4 items regarding “attaining/maintaining teacher registration” and “the National Quality Framework”. The third factor we named ‘Self’ (α = 0.67) which consisted of the two items regarding “Your professional self …e.g., passion and commitment to high quality ECE; intrinsic drive for quality improvement”. The Self factor was by far rated highest by respondents, followed by Workplace. These two factors were the only ones rated on average as “Agreed” (Workplace) or “Strongly agreed” (Self). The Requirements factor was on average not considered to motivate or support ECTs’ development and practice. The rotated factor matrix is presented in Table 3; descriptive statistics in Table 5. The extent to which ECTs felt motivated and supported by each of ‘self’, ‘workplace’ and ‘requirements’ were weakly interrelated (Table 6), meaning that ECTs’ experienced motivation and support from each was relatively independent of the others.

**Table 5:** Perceived motivators and supports for professional practice and benefits of teacher registration by State/Territory, location, service type, management structure and NQS rating (demographic categorical variables), among registered ECTs

		Self	Workplace	Requirements	Benefits
	*N*	*M* (*SD*)	*M* (*SD*)	*M* (*SD*)	*M* (*SD*)
*State/Territory*					
QLD	35	4.84 (0.38)	3.87 (0.84)	3.57 (0.82)	*3.36 (0.79)
NSW	238	4.58 (0.74)	3.79 (0.86)	3.33 (0.90)	2.80 (0.80)
VIC	62	4.56 (0.72)	3.75 (0.86)	3.11 (0.78)	2.92 (0.72)
SA	29	4.60 (0.79)	4.15 (0.81)	3.37 (1.04)	3.15 (0.65)
ACT	7	4.43 (0.79)	4.36 (0.48)	3.68 (0.51)	3.65 (0.68)
WA	4	4.13 (0.85)	3.56 (0.63)	2.75 (1.26)	2.58 (1.03)
TAS	3	4.83 (0.29)	3.58 (1.23)	3.50 (0.50)	3.02 (0.76)
NT	3	4.67 (0.58)	3.75 (0.25)	3.33 (0.77)	3.84 (0.47)
*Location*					
Metropolitan	248	4.60 (0.69)	3.83 (0.88)	3.32 (0.87)	2.93 (0.79)
Rural	133	4.59 (0.77)	3.83 (0.80)	3.34 (0.93)	2.90 (0.83)
^b^*Service type*					
Preschool/kindergarten	185	4.62 (0.67)	*4.01 (0.75)	3.39 (0.85)	*3.00 (0.83)
Long day care	171	4.59 (0.72)	3.62 (0.91)	3.24 (0.93)	2.82 (0.75)
*Management structure*					
Not for profit	93	4.63 (0.67)	*3.93 (0.78)	3.35 (0.84)	*2.96 (0.80)
For profit	288	4.50 (0.84)	3.52 (0.97)	3.25 (1.01)	2.79 (0.78)
*NQS rating*					
Not yet rated	27	4.65 (0.53)	3.67 (1.17)	3.53 (0.85)	2.81 (0.67)
Working towards	38	4.51 (0.94)	3.51 (0.92)	3.39 (0.94)	2.99 (0.94)
Meeting	125	4.52 (0.86)	3.68 (0.86)	3.23 (0.93)	2.84 (0.79)
^c^Exceeding/excellent	191	4.65 (0.56)	*4.01 (0.73)	3.34 (0.85)	2.98 (0.79)
^a^Totals	381				2.92 (0.80)

^a^Listwise *N*s

^b^25 of these ECTs were employed in ‘other’ settings

^c^Combined categories as only 4 ECTs worked at services rated ‘excellent’

^*^Denotes significantly higher scores per factor relative to comparison groups, reported in Sect. 4.5.1

**Table 6:** Spearman correlations between perceived benefits of teacher registration, supports for professional practice and demographic continuous variables among registered ECTs

	Benefits	Self	Workplace	Requirements	Provider type^a^	*n* ECTs	*n* children	Teacher registration^b^	Time registered
benefits	–								
self	.07	–							
workplace	.25^**^	.15^**^	–						
requirements	.60^**^	.15^**^	.25^**^	–					
provider type^a^	−.10^†^	.04	−.26^**^	−.09	–				
*n* ECTs	.00	−.07	.17^**^	−.04	−.09	–			
*n* children	−.04	−.11^*^	−.10^†^	−.07	.19^**^	.44^**^	–		
teacher registration^b^	.21^**^	.05	.07	.08	−.07	.07	−.15^**^	–	
time registered	.20^**^	.05	.07	.06	.01	−.04	−.04	.42^**^	–
time teaching	−.04	.10^†^	.03	.01	−.09	.02	−.22^**^	.39^**^	.18^**^

^**^p < .01, *p < .05, † *p* < .07; Listwise *N* = 353 registered ECTs

^a^Stand-alone service, one of up to 10 services managed by the same provider, one of up to 25 services managed by the same provider, one of more than 25 services managed by the same provider

^b^Conditional or provisional, proficient, highly accomplished or lead teacher

### General benefits of teacher registration

We similarly explored whether the items concerning perceived benefits of teacher registration (15 items) tapped into larger constructs for registered ECTs. Image factor analysis identified a single underlying factor supported by the scree-plot criterion, explaining 48% of the total variance, on which each item loaded significantly. We named this factor ‘Benefits’ (α = 0.93). On average, participants neither agreed nor disagreed that there were benefits to teacher registration. The factor matrix is presented in Table 4; descriptive statistics in Table 5. Perceived benefits of teacher registration correlated strongly with the extent to which ECTs perceived their professional development and practice improvement to be motivated and supported by ‘requirements’ (which was low rated overall), weakly with ‘workplace’, and unrelated to ‘self’ (Table 6).

### Contextual associations with motivators and supports for professional practice and perceived benefits of teacher registration

Next, we explored the extent to which ECTs’ reported motivators and supports for professional development and improved practice (‘self’, ‘workplace’ and ‘requirements’) and perceived benefits of teacher registration (‘benefits’) for registered participants depended on features of their employment context.

#### Associations with measured categorical demographics

A series of five MANOVAs compared the four factors according to measured categorical demographics: state/territory, location (metropolitan vs. rural; there were no remote), service type (preschool/kindergarten vs. long day care^4^), management structure (for profit vs. not for profit), and NQS rating. Descriptive statistics are reported in Table 5. There were no statistically significant differences between compared groups on either ‘self’ or ‘requirements’ as motivators and supports of ECTs’ development and practice. Thus, ECTs perceived their professional development and practice to be primarily driven by their ‘self’, and considered ‘requirements’ not to be relevant, regardless of their jurisdiction, location, service type, management structure or NQS rating. Four of the five MANOVAs showed significant multivariate effects (no factor differed significantly according to metropolitan or rural location), accounted for by group differences on ECTs’ perceptions of ‘workplace’ motivation and support for their professional practice and/or perceived ‘benefits’ of teacher registration, which each associated with certain contextual features of their employment.

There was a statistically significant multivariate effect of *states/territories* on reported motivators and supports for professional development and practice improvement, and perceived benefits of teacher registration (Pillai’s = 0.13, *F*(28, 1492) = 1.84, *η*_p_^2^ = 0.03, *p* = 0.005). This was due to a significant univariate effect on ‘benefits’ (*F*(7, 373) = 4.03, *η*_p_^2^ = 0.07, *p* < 0.001). Queensland respondents, from the only state/territory to adopt Approach 3 (refer Table 1), rated the perceived benefits of teacher registration higher than respondents from NSW (Tukey post hoc tests, *p* = 0.01; other states/territories scores were not significantly differently from Queensland or NSW).^5^

A significant multivariate effect of *service type* (Pillai’s = 0.06, *F*(4, 351) = 5.59, *η*_p_^2^ = 0.06, *p* < 0.001) was due to univariate effects on ‘workplace’ (*F*(1, 354) = 20.06, *η*_p_^2^ = 0.05, *p* < 0.001) and ‘benefits’ (*F*(1, 354) = 5.70, *η*_p_^2^ = 0.02, *p* = 0.018). ECTs in preschool/kindergarten settings rated ‘workplace’ motivators and supports for their development and practice as well as the perceived benefits of teacher registration, significantly higher than those working in long day care settings.

A significant multivariate effect of *management structure* (Pillai’s = 0.05, *F*(4, 376) = 4.71, *η*_p_^2^ = 0.05, *p* = 0.001) was again due to univariate effects on ‘workplace’ (*F*(1, 379) = 16.76, *η*_p_^2^ = 0.04, *p* < 0.001) and ‘benefits’ (*F*(1, 379) = 4.02, *η*_p_^2^ = 0.01, *p* = 0.046). ECTs working in not-for-profit services rated ‘workplace’ and ‘benefits’ significantly higher than those working at for profit services.

The final significant multivariate effect of *NQS rating* (Pillai’s = 0.07, *F*(9, 1290) = 3.17, *η*_p_^2^ = 0.02, *p* = 0.001) was due to a univariate effect on ‘workplace’ (*F*(3, 430) = 8.65, *η*_p_^2^ = 0.06, *p* < 0.001). ECTs working in services rated as exceeding NQS or excellent, rated ‘workplace’ motivators and supports for their professional development and practice improvement significantly higher than ECTs working in services rated as ‘meeting NQS’, ‘working towards NQS’ or ‘not yet rated’ (Tukey post hocs, *p*s < 0.01).

#### Associations with measured continuous demographics

Spearman correlations measured the extent to which registered ECTs’ reported motivators and supports for professional development and practice improvement (‘self’, ‘workplace’ and ‘requirements’) and perceived benefits of teacher registration were associated with measured demographic continuous variables: provider type (stand-alone or one of several services managed by the same provider), number of ECTs in the workplace, number of children for which the service was licensed per day, level of ECTs’ registration, length of time they had been registered, and total length of teaching experience. Statistics are reported in Table 6.

Perceived ‘workplace’ support (i.e., fellow educators and employers) for registered ECTs’ reported motivators and supports of professional development and practice improvement associated positively albeit weakly, with the number of ECTs employed at their workplace; and associated negatively but weakly, with the number of services managed by ECTs’ workplace provider and number of children for which they were licensed. The extent to which ECTs perceived their ‘self’ to drive professional development and practice associated negatively and weakly with the number of children for whom their service was licensed, and there was a trend towards a positive but weak relationship with their total length of teaching experience. There were no significant correlations between the demographic variables and the extent to which ECTs perceived ‘requirements’ (i.e., NQF and teacher registration) to motivate and support their professional development and practice improvement.

ECTs who held higher levels of registration perceived greater ‘benefits’ to teacher registration, as did ECTs who had been registered for longer. There was a trend towards ECTs who worked in one of several services managed by the same provider to perceive lower benefits to teacher registration.

Among the demographics themselves, working at one of several services managed by a same provider correlated with licensing for more children. Services licensed for more children employed more ECTs who were unregistered or registered at lower levels and had less teaching experience. ECTs registered for longer had more teaching experience and were registered at higher levels.

### Teacher registration policy directions

#### Registered ECTs

Recommendation 5 of AITSL’s teacher registration review (Education Services Australia, 2018) was that, irrespective of their place of employment, all ECTs in Australia be required to register under a consistent national system alongside primary and secondary school teachers. In our study, this policy recommendation attracted an overall mean rating of just below 4 (i.e., just below “Agree”). Respondents overall also rated just below 4, the recommendation that instead of a national system of teacher registration, government funding should be used to provide ECTs with ongoing access to quality professional development and mentoring. What respondents most supported on average was a nationally consistent system of teacher registration that provided ECTs with funding to access quality professional development and mentors to support their transition from provisional to proficient, rated above 4 on the scale in all states/territories. There were no significant differences in ratings according to states/territories across the three options (see Table 7).

**Table 7:** Extent of registered ECTs’ agreement with recommendation 5 in AITSL’s teacher review

	Whole sample	QLD	NSW	VIC	SA	ACT	WA	TAS	NT
	*N* = 406	*n* = 32	*n* = 231	*n* = 61	*n* = 30	*n* = 5	*n* = 3	*n* = 3	*n* = 3
	*M (SD)*	*M (SD)*	*M (SD)*	*M (SD)*	*M (SD)*	*M (SD)*	*M (SD)*	*M (SD)*	*M (SD)*
I would like all ECTs in Australia who work with children in the birth—5 years age group register under a consistent national system with primary and secondary school teachers	3.86 (1.15)	4.03 (0.97)	3.72 (1.22)	4.11 (1.00)	3.97 (0.93)	4.80 (0.44)	3.50 (1.73)	4.33 (0.58)	4.67 (0.58)
Instead of introducing a national system of teacher registration for ECTs working with children in the birth—5 years age group, I would like government funding to be used to provide ECTs with ongoing access to quality professional development and men	3.88 (1.12)	3.88 (1.04)	3.89 (1.15)	3.90 (1.14)	3.87 (0.97)	4.00 (1.00)	4.25 (0.96)	3.67 (0.58)	2.00 (1.00)
I would like a national system of registration for ECTs introduced that provides ECTs with funding to access quality professional development and mentors to support ECTs’ transition from provisional to proficient	4.28 (0.80)	4.28 (0.77)	4.32 (0.81)	4.20 (0.87)	4.10 (0.71)	4.80 (0.45)	4.50 (0.58)	4.33 (0.58)	4.00 (1.00)

All items were rated on a 5-point scale from 1: Strongly disagree, 2: Disagree, 3: Neither agree nor disagree, 4: Agree, 5: Strongly agree

There was no significant difference across states/territories, Pillai’s Trace = .08, *F*(21, 1083) = 1.40, *η*_p_^2^ = .026, *p* = .11. Listwise *N*s are reported

If a nationally consistent system of registration for ECTs were to be implemented, respondents were asked to provide their level of agreement with each of seven options as to how this system could operate. Means and standard deviations are reported in Table 8; with results showing no significant differences in ratings according to states/territories across the seven options. The options that were most endorsed, receiving a mean score of 4 (“Agree”; rounded to the nearest scale point) were Option 5: “The system is developed and overseen by AITSL and ACECQA, for all teachers (i.e., early childhood, primary and secondary) with revised professional teaching standards that reflect the expertise, roles and responsibilities of ECTs who work with children in the birth-5 years age group” (*M* = 3.62, SD = 1.25), and Option 4: “The system is developed and overseen by AITSL for all teachers (i.e., early childhood, primary and school teachers) with revised professional teaching standards that reflect the expertise, roles and responsibilities of ECTs who work with children in the birth—5 years age group” (*M* = 3.59, SD = 1.20). Respondents least supported Options 1 and 2, referring to a separate system of registration just for ECTs who worked with children in the birth—5 years age group, irrespective of whether registration was embedded into the NQF or was developed and overseen by ACECQA. None of the options was “strongly agreed”, on average.

**Table 8:** Extent of registered ECTs’ agreement with alternative options for the implementation of AITSL’s recommendation 5

	Whole sample	QLD	NSW	VIC	SA	ACT	WA	TAS	NT
	*N* = 364	*n* = 31	*n* = 228	*n* = 61	*n* = 29	*n* = 5	*n* = 4	*n* = 3	*n* = 3
	*M (SD)*	*M (SD)*	*M (SD)*	*M (SD)*	*M (SD)*	*M (SD)*	*M (SD)*	*M (SD)*	*M (SD)*
OPTION 1: The system is developed and overseen by ACECQA, is only for ECTs who work with children in the birth—five age group (not teachers who work with primary or school aged children), and is integrated into the NQF’s Assessment	2.81 (1.25)	2.55 (1.26)	2.79 (1.33)	2.95 (1.22)	3.03 (1.38)	1.80 (1.10)	4.00 (1.41)	2.00 (1.00)	2.33 (1.53)
OPTION 2: The system is developed and overseen by ACECQA, is only for ECTs who work with children in the birth—five group (not teachers who work with primary or school aged children), and is separate to the NQF, with ECTs required to adhere to professional teaching standards that are specific to teachers who work with children in the birth—5 years age group	2.79 (1.21)	2.55 (1.18)	2.79 (1.21)	3.05 (1.10)	2.83 (1.37)	1.80 (1.10)	3.00 (1.41)	2.33 (1.53)	2.00 (1.00)
OPTION 3: The system is developed and overseen by Early Childhood Australia, is only for ECTs who work with children in the birth—five age group (not teachers who work with primary or school aged children), with ECTs required to adhere to professional teaching standards that are specific to teachers employed to work with children in the birth—5 years age group	3.09 (1.28)	2.71 (1.32)	3.14 (1.27)	3.33 (1.19)	2.79 (1.21)	2.00 (1.41)	3.75 (1.50)	2.33 (1.53)	2.33 (1.53)
OPTION 4: The system is developed and overseen by AITSL for all teachers (i.e. early childhood, primary and school teachers) with revised professional teaching standards that reflect the expertise, roles and responsibilities of ECTs who work with children in the birth—5 years age group	3.59 (1.20)	4.06 (1.12)	3.42 (1.23)	3.72 (1.11)	3.86 (1.03)	4.20 (0.84)	3.75 (1.89)	4.67 (0.58)	3.67 (1.53)
OPTION 5: The system is developed and overseen by AITSL and ACECQA, for all teachers (i.e. early childhood, primary and school teachers) with revised professional teaching standards that reflect the expertise, roles and responsibilities of ECTs who work with children in the birth—5 years age group	3.62 (1.25)	3.81 (1.17)	3.43 (1.29)	4.07 (1.03)	3.69 (1.20)	4.60 (0.89)	3.50 (1.73)	4.67 (0.58)	4.00 (1.00)
OPTION 6: The system is developed and overseen by AITSL for all teachers (i.e. early childhood, primary and school teachers) but with separate professional teaching standards i.e. separate standards for teachers working with children in the birth—5 years age group and teachers working with school aged children	3.26 (1.19)	3.45 (1.18)	3.18 (1.23)	3.46 (1.13)	3.31 (0.89)	3.80 (1.64)	2.75 (1.50)	3.00 (1.00)	3.33 (0.58)
OPTION 7: The system is developed and overseen by AITSL and ACECQA, for all teachers (i.e. early childhood, primary and school teachers) but with separate professional teaching standards i.e. separate standards for teachers working with children in the birth—5 years age group and teachers working with school aged children	3.17 (1.23)	3.19 (1.45)	3.08 (1.22)	3.49 (1.22)	3.10 (1.08)	3.40 (1.34)	4.00 (0.82)	3.00 (1.00)	3.00 (1.00)

All items were rated on a 5-point scale from 1: Strongly disagree, 2: Disagree, 3: Neither agree nor disagree, 4: Agree, 5: Strongly agree

There was no significant difference across states/territories, Pillai’s Trace = .17, *F*(49, 2492) = 1.27, *η*_p_^2^ = .024, *p* = .10. Listwise *N*s are reported

#### Unregistered ECTs

Non-registered ECTs’ responses mostly mirrored their registered colleagues’. Differences were apparent for “I would like a national system of registration for ECTs introduced that provides ECTs with funding to access quality professional development and mentors to support ECTs’ transition from provisional to proficient”, more preferred by non-registered than registered ECTs (see Table 9). Among the 7 presented potential policy options, registered ECTs preferred Option 4 more than non-registered ECTs (“The system is developed and overseen by AITSL for all teachers (i.e. early childhood, primary and school teachers) with revised professional teaching standards that reflect the expertise, roles and responsibilities of ECTs who work with children in the birth—5 years age group”; *M* = 3.17, SD = 1.11), whereas non-registered ECTs preferred Option 7 more than registered ECTs (“The system is developed and overseen by AITSL and ACECQA, for all teachers (i.e. early childhood, primary and school teachers) but with separate professional teaching standards i.e. separate standards for teachers working with children in the birth—5 years age group and teachers working with school aged children”; *M* = 3.64, SD = 3.17).

**Table 9:** Extent of registered vs. non-registered ECTs’ agreement to recommendation 5 in AITSL’s review, and options for its implementation

	Registered	Non-registered
	*n* = 364	*n* = 36
	*M* (*SD*)	*M* (*SD*)
I would like all ECTs in Australia who work with children in the birth—5 years age group register under a consistent national system with primary and secondary school teachers	3.86 (1.15)	3.67 (1.12)
Instead of introducing a national system of teacher registration for ECTs working with children in the birth—5 years age group, I would like government funding to be used to provide ECTs with ongoing access to quality professional development and men	3.88 (1.12)	4.14 (1.08)
^a^I would like a national system of registration for ECTs introduced that provides ECTs with funding to access quality professional development and mentors to support ECTs’ transition from provisional to proficient	4.28 (0.80)	4.61 (0.77)
OPTION 1: The system is developed and overseen by ACECQA, is only for ECTs who work with children in the birth-five age group (not teachers who work with primary or school aged children), and is integrated into the NQF’s Assessment	2.81 (1.25)	2.86 (1.33)
OPTION 2: The system is developed and overseen by ACECQA, is only for ECTs who work with children in the birth-five group (not teachers who work with primary or school aged children), and is separate to the NQF, with ECTs required to adhere to professional teaching standards that are specific to teachers who work with children in the birth-5 years age group	2.79 (1.21)	3.14 (1.20)
OPTION 3: The system is developed and overseen by Early Childhood Australia, is only for ECTs who work with children in the birth-five age group (not teachers who work with primary or school aged children), with ECTs required to adhere to professional teaching standards that are specific to teachers employed to work with children in the birth-5 years age group	3.09 (1.28)	3.42 (1.18)
^b^OPTION 4: The system is developed and overseen by AITSL for all teachers (i.e. early childhood, primary and school teachers) with revised professional teaching standards that reflect the expertise, roles and responsibilities of ECTs who work with children in the birth-5 years age group	3.59 (1.20)	3.17 (1.11)
OPTION 5: The system is developed and overseen by AITSL and ACECQA, for all teachers (i.e. early childhood, primary and school teachers) with revised professional teaching standards that reflect the expertise, roles and responsibilities of ECTs who work with children in the birth-5 years age group	3.62 (1.25)	3.72 (1.16)
OPTION 6: The system is developed and overseen by AITSL for all teachers (i.e. early childhood, primary and school teachers) but with separate professional teaching standards i.e. separate standards for teachers working with children in the birth-5 years age group and teachers working with school aged children	3.26 (1.19)	3.28 (1.09)
^b^OPTION 7: The system is developed and overseen by AITSL and ACECQA, for all teachers (i.e. early childhood, primary and school teachers) but with separate professional teaching standards i.e. separate standards for teachers working with children in the birth-5 years age group and teachers working with school aged children	3.17 (1.23)	3.64 (1.18)

All items were rated on a 5-point scale from 1: Strongly disagree, 2: Disagree, 3: Neither agree nor disagree, 4: Agree, 5: Strongly agree

^a^There was a significant multivariate difference in registered vs. unregistered ECTs’ agreement to recommendation 5 from AITSL’s review, Pillai’s Trace = .02, *F*(3, 401) = 2.78, *η*_p_^2^ = .02, *p* = .041. This was due to non-registered ECTs’ greater endorsement of the third item, *F*(1, 403) = 5.57, *η*_p_^2^ = .01, *p* = .019

^b^There was a significant multivariate difference in registered vs. unregistered ECTs’ agreement across the 7 options for implementation of AITSL’s recommendation 5, Pillai’s Trace = .04, *F*(7, 392) = 2.14, *η*_p_^2^ = .04, *p* = .039. This was due to registered ECTs’ greater endorsement of Option 4, *F*(1, 398) = 4.01, *η*_p_^2^ = .01, *p* = .046, and non-registered ECTs’ greater endorsement of Option 7, *F*(1, 398) = 7.11, *η*_p_^2^ = .01, *p* = .036

Collectively, these findings suggest that support for a nationally consistent system of teacher registration that included early childhood, primary and secondary teachers, is limited. Support would be strengthened, however, if such a policy included access to quality professional development and mentors for graduate ECTs. Additional measures that promote the specialist expertise of ECTs could also strengthen the efficacy of such a system. These measures align with recommendation 6 of the national review (Education Services Australia, 2018), that the APST be revised so that they are more relevant and applicable to ECTs, and include the involvement of ACECQA, the regulatory body that oversees the NQF for ECE services.

## Discussion

This study contributes new understandings and empirical evidence regarding registered ECTs’ perceptions of current and proposed teacher registration policy in Australia, contextualised against other motivators and supports, and characteristics of their workplace settings. More broadly, the study contributes to the growing body of literature that disrupts discursive truths that claim accountability systems support teacher quality.

Our findings problematise the governing of ECTs through teacher registration. For this study’s participants, teacher registration did not drive their professional development or practice improvement, nor did they on average agree that teacher registration improved the quality of ECEC services, the quality of their own practice (in terms of their professional development and practice improvement), outcomes for children, or ECTs’ professional status. Participants only agreed that teacher registration added to regulatory burden and disagreed that it increased their salary. These reported perceptions and experiences call into question policy discourses in Australia (Education Services Australia, 2018) and internationally (OECD, 2013) that purport standards regimes such as teacher registration as necessary and effective policy levers to improve education standards.

While ECT respondents overall neither agreed nor disagreed with purported truth claims about the affordances of teacher registration, of note was their highlighting of themselves as the strongest motivator and support for their professional development and improvement of practice. For these ECTs, intrinsic supports and motivators—passion, commitment to high-quality ECE, and a drive to improve practice—and not extrinsic regulatory requirements, most influenced teaching quality. This is a notable finding that challenges the deficit positioning of ECTs and teachers more broadly in current policy in Australia (Barnes, 2021). The finding also problematises a premise of teacher registration, that teachers cannot be trusted to provide quality teaching and take responsibility for their professional development (Chatelier & Rudolph, 2018). In contrast to top-down managerial approaches to professionalism (Havnes, 2018; Oberhuemer, 2005), these ECTs’ subjectivities appear grounded in professional autonomy and expert knowledge rather than performativity.

Policy effects are key concerns of critical policy sociologists, in particular, how “policy forms the objects of which it speaks” (Ball, 2021, p. 2). Our findings appear to suggest that the lived experience of teacher registration for ECT respondents—its generally limited affordances for them and for the sector –led them to reject discursive truths about the need for and benefits of teacher registration. Moreover, as agents rather than policy subjects, these ECTs generally appear to be actively engaging with and negotiating registration discourses to support their own construction of professional teachers. Not only did respondents not reach overall agreement with the Review recommendation that a nationally consistent system of teacher registration be developed for early childhood, primary and secondary teachers, clear support was shown in each state/territory jurisdiction, as well as across registered and non-registered ECTs, only for the proposal that such a system be introduced with funding for professional development and mentoring. This support is consistent with the study’s finding that ECTs are intrinsically motivated to undertake professional development and improve their practice. As providers can find investment in professional development difficult (ACECQA, 2019), undertaking such development can be an impost on ECTs’ own time and expense (Fenech et al., 2021).

Given that the situational context of teachers affects their engagement with policy (Braun et al., 2010), we considered registered respondents’ demographic and employment contexts as potential influences on the study’s findings. That teacher registration was not regarded as a driver of professional development or practice improvement, and ECT respondents overall neither agreed nor disagreed with purported truth claims about the affordances of teacher registration, could in part be explained by the fact that an overwhelming majority of respondents (93%) were employed in non-school settings. As such, they may have been less amenable to prevailing discourses about teaching standards and teacher registration disseminated by AITSL. This hypothesis is supported by other findings from our study. First was that Queensland registered respondents—most of whom were employed in schools—rated the benefits of teacher registration significantly higher (although still below Agree) than NSW registered respondents, most of whom were employed in non-school settings. Second, respondents employed in preschools/kindergartens rated registration benefits higher than teachers employed in long day care (although still below Agree), a finding that could be attributed to preschools in some jurisdictions being predominately operated by state/territory governments (ACT, Northern Territory, South Australia, Western Australia). Unsurprisingly, higher levels (highly accomplished; lead) and longer periods of registration correlated (albeit weakly) with higher perceived benefits.

In the remainder of the paper we consider the implications of our findings in light of policy developments pertaining to teacher registration that have emerged since the administration of our survey. Following our cohabitating (Salomon, 1990) of quantitative findings with critical social policy theorising, our discussion is grounded in a consideration of policy (teacher registration) as a discursive representation of particular values (Regmi, 2019) and, consistent with our values as education researchers, a critique of “neoliberal forces on education policies and practices” (Regmi, p. 66). This approach aligns with the critical policy sociology tradition of making explicit the values implicit in policy *and* those held by policy researchers. We also take up Rizvi and Lingard’s challenge that in addition to critique, critical policy sociology should “also point to strategies for progressive change” (2010, p. 51).

### Future policy directions

Since the collection of data for this study, moving to a nationally consistent system of teacher registration for all teachers—irrespective of whether they are employed in school or non-school settings—is being pursued as a workforce strategy to improve the professional recognition of ECTs (ACECQA, 2021a). In a developing 10-year national early childhood workforce strategy, stakeholder consultation was sought on multiple strategies to address longstanding ECT and educator shortages. Teacher registration as a strategy to improve professional recognition was supported or strongly supported by 85% of all survey respondents (ACECQA, 2021b), and by 91% of the 359 respondents who identified that their main role was as an ECT (personal communication).

This national workforce strategy finding, undertaken approximately 18 months after the teacher registration survey reported in this paper was administered, provides further insight into how the limited support for teacher registration identified in our study might be interpreted. Central to this understanding is the different situating of teacher registration in the two surveys. As noted above, the ACECQA survey was undertaken to identify support for strategies to address attraction, supply, and retention workforce. Accordingly, teacher registration was positioned as a potential strategy to support ECTs’ professional recognition. In contrast, the focus on teacher registration in our study was primarily as a possible driver of teacher quality. Notwithstanding potential differences in the two ECT survey respondents, supporting teacher registration in the ACECQA workforce survey as a solution to the longstanding issues of low pay and professional status, but not in our study as a strategy to improve teacher quality (unless access to quality professional development and mentoring is embedded into the system), may be illustrative of ECTs exercising agency in an era of increasing accountability. It is conceivable that while ECTs may not view teacher registration as a means to improve their teaching practice (again, unless professional development and mentoring is provided), that at a time when government policy has turned its attention to workforce shortages, they may strategically support teacher registration in a political climate that is seemingly more conducive to improving their pay and working conditions. Being judicious about the affordances of teacher registration policy may be indicative of ECTs as policy actors, rather than teacher registration policy subjects (Ball et al., 2011; Barnes, 2021).

Such a strengths-based positioning of ECTs is, however, complicated by the perception that the greatest affordance of professional registration is its potential to improve the professional status of the ECT workforce. ECT advocates have long fought for professional recognition and pay parity with teachers in schools, yet still today, ECTs employed in non-school settings can earn up to $30,000 less a year than teachers employed in schools (Independent Education Union of Australia, no date). Consideration of this historical context highlights how a reliance on teacher registration for professional recognition and pay parity, and indeed for greater access to professional development and mentoring, is potentially dangerous. Such a dependence on registration policy may be indicative of ECTs as policy subjects who accept the logics of teacher registration and ironically, pursue “the [very] regime of accountability … [that] breed[s] a culture of mistrust in which the teacher must continuously prove themselves worthy of their place within the profession” (Chatelier & Rudolph, 2018, p. 9).

The production of such policy subjects is a discursive effect of what critical policy sociologists (e.g., Bacchi, 2009; Ball, 2021) remind us is a selective choice by governments about which issues constitute policy problems and which do not. In the case of teacher registration, we contend that teacher-subjects have emerged not only from an intensification of teacher and teacher education accountability technologies, but from persistent policy inattention to three structural issues that collectively impact the education quality of the ECE sector. First, and as previously mentioned, is ECTs’ low professional pay and status, a longstanding contributor to ECT turnover and undersupply (Fenech & King, 2020). Second, and consistent with dominant policy approaches internationally (Robert-Holmes & Moss, 2021), is the marketisation of ECE (Fenech, 2019). Currently in Australia, two-thirds of long day care services are for-profit (ACECQA, 2021a), yet it is for-profit services that have consistently been shown to be operating below national quality standards (Fenech, 2019). Third are regulatory requirements pertaining to the employment of ECTs, which have resulted in ECTs often being the only teacher employed in their service (Education Services Australia, 2018). Our findings show that perceived workplace support for teaching practice and professional development from ECTs’ colleagues and employer was greater in settings that employed more ECTs, and in services that were preschools, not-for-profit, and had an NQS exceeding or excellent quality rating. Moreover, ECTs’ professional selves was the greatest perceived support to their teaching practice and professional development, regardless of workplace setting. In light of these findings, policy attention to the three identified structural barriers to quality ECE seemingly would support quality teaching and early education, and potentially mitigate the need for teacher registration. Given the impending introduction of a new national system of teacher registration, however, our findings caution that without attention to these structural quality barriers, the impact of a registration system on teacher quality and quality early education may be limited.

### Towards national teacher registration

In the context of entrenched professional marginalisation and a developing national early childhood workforce strategy that endorses a national system of registration inclusive of ECTs, moving forward, how might ECTs be policy actors, not policy subjects, in such a landscape? While critical policy sociology should, in addition to critique, “also point to strategies for progressive change” (Rizvi & Lingard, 2010, p. 51), given the inevitability of a national registration system being introduced, in the remainder of the paper we pragmatically consider “how can we reclaim teacher evaluation [registration] so that it supports teachers?” (Garver, 2019, p. 19). To address these questions, we propose interdependent policy directions specific to three levels of governance: standards governance (the registration system itself); service governance (employers of ECTs); and self-governance (ECTs taking responsibility for their own professionalism).

#### Standards governance

To overcome the reported lived experience of registration not supporting teachers’ practice or benefitting the ECE sector, and thus potentially being perceived only as a surveillance mechanism (Garver, 2019; Havnes, 2018), a new national system will need to promote a strengths rather than deficit positioning of ECTs and the work they do. This approach requires a view of ECTs as professionals, not technicians, with specialist knowledge and skills, capable of exercising autonomy and making judgements that support children’s learning and development. It seems feasible to suggest that if ECTs perceived that teacher registration bodies considered them to be competent professionals with a right to ongoing support to enhance their professionalism, then a system of teacher registration would likely engender stronger support.

To this end, ECTs should have input into the development of professional teaching standards that are relevant to and reflect the complexity of their practice. While in Australia and internationally teaching standards are generally externally determined (Havnes, 2018), the development of a progressive system of teacher registration in Australia requires a professionalism approach “from within” rather than “from above” (Havnes, 2018, p. 670). This strategy is critical to the future authentic inclusion of ECTs in teacher registration, given that the previous review of Australia’s teaching standards (AITSL, 2016) was conducted 2013–2015, before the majority of ECTs were required to be professionally registered, and the 2018 Review recommended amendments to the Standards “to ensure their relevance and applicability to ECTs” (Education Services Australia, 2018, p. v).

In addition to amending the Standards for teachers, as endorsed by respondents in our study (and particularly by non-registered ECTs), a new national system of teacher registration will need to encompass more effective provisioning of quality professional development, and better access to mentors, especially for ECTs employed in non-school settings. While the Review proffered the theme of ‘one profession’ (Education Services Australia, 2018), ECTs employed in non-school settings work in a landscape that contrasts starkly with teachers employed in schools. In addition, as noted earlier, ECTs are often the only qualified teacher employed in a non-school setting (Education Services Australia, 2018). Regulatory reform must therefore take into account not only ECTs, but ECTs in context.

#### Service governance

A key finding from our study is that ECTs considered their colleagues and employers to support their provision of quality education and professional development. What is not clear, however, is what this support looks like in practice, and whether this support would enable ECTs to meet registration requirements. As noted earlier, while there is a growing body of research that has explored the enactment and effects of teacher accountability policies in schools (e.g., Ball et al., 2011; Chatelier & Rudolph, 2018; Garver, 2019) a strong evidence-base from non-school early childhood settings is lacking. Future research is needed to inform understandings about the extent to which early childhood service providers engage with teacher registration, and what policy translation and enactment (Braun et al., 2010) look like in these services.

The teacher registration review recommended that “teacher employers maintain responsibility and strengthen their role in providing access for ECTs to high quality induction and mentoring, to support their transition into the workplace and the profession” (Education Services Australia, 2018, p. iii). Given the variability of non-school early childhood service providers, the quality provided, the prevalence of for-profit providers, and the shortage of ECTs (ACECQA, 2021a) this recommendation is unlikely to be implemented consistently across the sector, particularly if responsibility extends to providing access to quality professional development. In its effects, this policy recommendation shifts attention away from an aforementioned entrenched barrier to the provisioning of quality early childhood education: the marketisation of the sector and ensuing for-profit provisioning (Fenech, 2019). Without a registration system in which access to mentoring and professional development for ECTs is embedded—the preferred way forward for ECTs in this study—the effective implementation of such a recommendation seems unlikely.

#### Self governance

Our study demonstrates that ECTs can and do take responsibility for their own professionalism, not to meet externally imposed registration requirements, but to be effective teachers. With a policy trajectory intent on issuing a new national registration system, it will be critical that ECTs preserve and nurture this professionalism-from-within and utilise this frame to evaluate the utility of externally imposed professionalism. Consistent with our findings that colleagues supported ECTs’ quality teaching and professional development, resisting potential deleterious policy effects will also require the forging of collegial relationships and communities of practice (Garver, 2019). As maintained earlier, advocating for recognition of specialist expertise and scope to exercise professional autonomy (Havnes, 2018) will also be critical, albeit we argue that this advocacy needs to transcend to activism for policy to redress long-ignored systemic barriers to teacher quality, in particular, low pay and professional status, for-profit provisioning of ECE, and limited requirements for the employment of ECTs in ECE services.

## Conclusion

Critical policy sociology applied conceptually and without attention to how policy effects are experienced, risks limited potency to engender policy that is transformative (Hunkin et al., 2020). Mindful of this caution, our study investigated ECTs’ perceptions of teacher registration and a recommendation to introduce a nationally consistent system of registration for all teachers in Australia. Situating this study in a broader investigation of ECTs’ perceived motivators and supports for professional development and practice improvement allowed for a problematising of teacher registration that affirms and extends extant theoretical analyses and empirical research that are school (primary and secondary) focused. Current fragmented approaches to ECT registration were overall not considered to motivate or support professional development or practice improvement, and on average, there was neither agreement nor disagreement that teacher registration benefitted ECTs or the ECE sector. While our study showed support for a registration system that embedded funding for professional development and mentoring, that ECTs’ practice and development was most driven by intrinsic motivation and, to a lesser extent, being employed in high-quality, not-for-profit, and preschool settings where other ECTs are employed, suggests that more effective and progressive policy approaches require an addressing of the marketisation of the sector, ECTs’ longstanding low pay, and regulations pertaining to the employment of ECTs.

## Footnotes

1 These teachers may be graduates of birth-five, birth-eight, or birth-12 initial teacher education degree programmes. While internationally ECE is commonly regarded as birth-eight years, in this paper we confine our focus to birth-five years and thus to teachers who work with young children prior to formal schooling. These ECTs can be employed in school or prior-to-school settings (e.g., long day care; community or Council preschools).

2 The National Quality Framework is a system of regulation, quality assurance, and quality improvement for early childhood education and care services in Australia. The Framework consists of National Quality Standards (NQS) against which services are assessed and rated.

3 Participants selected one of nine provided age-ranges: 18–25, 26–30, 31–35, 36–40, 41–45, 46–50, 51–55, 56–60, Over 60 (see Table 2).

4 Preschools/kindergartens generally operate during school terms and times, for children aged 3–5 years. Long day care services provide early learning and care for children aged birth-five years generally from 7am to 6 pm to cater for working parents. Long day care services may operate preschool programmes, that is, educational programmes run by an ECT for 15 h per week, 40 weeks of the year, for children in the year prior to starting school.

5 Due to low numbers in some states/territories, a subsequent MANOVA was estimated including only Queensland, NSW, Victoria and South Australia. This also showed a significant multivariate effect of State (Pillai’s Trace = .09, *F*(12, 1077) = 2.69, *η*_p_^2^ = .029, *p* = .001) due to a significant difference on ‘benefits’ (*F*(3, 360) = 5.58, *η*_p_^2^ = .044, *p* = .001) rated higher by ECTs from Queensland than NSW (Tukey post hoc tests *p* = .002), and a trend for Queensland ECTs to rate the benefits of teacher registration higher than Victoria (*p* = .059). ECTs from South Australia scored in between and did not differ significantly from other states.
